# Comparison of Individual and Combined Effects of Four Endocrine Disruptors on Estrogen Receptor Beta Transcription in Cerebellar Cell Culture: The Modulatory Role of Estradiol and Triiodo-Thyronine

**DOI:** 10.3390/ijerph13060619

**Published:** 2016-06-22

**Authors:** Gergely Jocsak, David Sandor Kiss, Istvan Toth, Greta Goszleth, Tibor Bartha, Laszlo V. Frenyo, Tamas L. Horvath, Attila Zsarnovszky

**Affiliations:** 1 Department of Physiology and Biochemistry, Szent Istvan Faculty University of Veterinary Sciences, Budapest 1078, Hungary; kiss.david@aotk.szie.hu (D.S.K.); toth.istvan@aotk.szie.hu (I.T.); goszleth.greta@aotk.szie.hu (G.G.); bartha.tibor@aotk.szie.hu (T.B.); frenyo.laszlo@aotk.szie.hu (L.V.F.); zsarnovszky.attila@mkk.szie.hu (A.Z.); 2 Section of Comparative Medicine, Yale University School of Medicine, New Haven, CT 06520, USA; tamas.horvath@yale.edu; 3 Department of Animal Physiology and Animal Health, Szent Istvan Faculty University of Agriculture and Environmental Sciences, Godollo 2100, Hungary

**Keywords:** estrogen receptor beta, transcription, bisphenol A, zearalenone, arsenic, camphor, combined treatment

## Abstract

*Background*: Humans and animals are continuously exposed to a number of environmental substances that act as endocrine disruptors (EDs). While a growing body of evidence is available to prove their adverse health effects, very little is known about the consequences of simultaneous exposure to a combination of such chemicals; *Methods*: Here, we used an *in vitro* model to demonstrate how exposure to bisphenol A, zearalenone, arsenic, and 4-methylbenzylidene camphor, alone or in combination, affect estrogen receptor β (ERβ) mRNA expression in primary cerebellar cell cultures. Additionally, we also show the modulatory role of intrinsic biological factors, such as estradiol (E2), triiodo-thyronine (T3), and glial cells, as potential effect modulators; *Results*: Results show a wide diversity in ED effects on ERβ mRNA expression, and that the magnitude of these ED effects highly depends on the presence or absence of E2, T3, and glial cells; *Conclusion*: The observed potency of the EDs to influence ERβ mRNA expression, and the modulatory role of E2, T3, and the glia suggests that environmental ED effects may be masked as long as the hormonal milieu is physiological, but may tend to turn additive or superadditive in case of hormone deficiency.

## 1. Introduction

Endocrine disruptors (EDs) are environmental pollutants, phytoestrogens or mycoestrogens that, in case of exposure, disrupt the physiological regulatory pathways of some endogenous hormones, mostly those of estrogens (17β-estradiol, E2) and thyroid hormones (THs). The endocrine disrupting potency of EDs is, for the most part, the result of their ability to bind to either/or estrogen receptors (ERs) and TH receptors (TR). During their everyday lives, both humans and animals are simultaneously exposed to a multitude of EDs [1]. The vast majority of the toxicology studies; however, present data from single exposure experiments. Therefore, the potential cellular consequences of combined ED effects (simultaneous exposure) are not known.

In the present experiments, we were focusing on four potential EDs: bisphenol A, zearalenone, arsenic, and 4-methylbenzylidene camphor (alone and in combination). Bisphenol A (BPA) is used in the manufacture of certain plastics and epoxy resins, including a variety of common consumer goods, such as water bottles, sports equipment, CDs, and DVDs; epoxy resins containing BPA are used to line water pipes, as coatings on the inside of many food and beverage cans and in making thermal paper such as that used in sales receipts. Zearalenone (Zea), also known as F-2 mycotoxin, is a product of fusarium molds; Zea is heat-stable and is found worldwide in a number of cereal crops, such as maize, barley, oats, wheat, rice, and sorghum, and also in bread. Arsenic (As) contamination of groundwater is a problem that affects millions of people across the world. Camphor (4-methylbenzylidene camphor, MBC) is a natural compound in some tree species, rosemary leaves, *etc.*, and it is also synthetically produced by the industry, it is used for its scent, as UV filters, as an ingredient in cooking, as an embalming fluid, for medicinal purposes, and in religious ceremonies.

Estrogens (mostly 17β-estradiol, E2) and thyroid hormones (THs) play critical roles in the regulation of cerebellar development [2,3,4]. Estrogen receptors (ERs) are expressed in the cerebellum from early ages (postnatal period in rats) in a developmentally regulated fashion [2,5,6]. Likewise, THs also target just about all cell types in the cerebellum [7,8]. In addition to single or combined hormone effects, we also showed that the astroglia (glia) may be an additional site of these actions [9]. Based on the available literature and our studies, endocrine disruptor- (ED) induced disturbance of the physiological synchrony between E2 and THs during cerebellar development could be more pervasive and far-reaching than currently appreciated, and merits investigation [9]. Abnormalities in the hormonal milieu or in the expression and function of ERs (and TRs) result in deviations in the normal development of this brain area. For example, in individuals with hypothyroidism the migration and terminal differentiation of granule cell precursors could be impaired [8]. Developmental abnormalities can also lie on the grounds of altered glia differentiation or due to insufficient TR-ligand interactions [10,11,12]. Available data suggest that E2 and THs are equally important regulators of cerebellar development [13,14]. ERα,β play crucial roles in neural tissue development, with ERα being expressed in the fetal brain and during early postnatal days and ERβ persisting life-long in most cerebellar cell types [5]. Reports by Kirby *et al.* [15] support the idea that E2 stimulates cell migration through ERs. Knock-out of ERβ leads to several morphological alterations in the cerebellar tissue, most likely due to the lack of estrogenic effects in the regulation of cell migration [16]. Thus, ERβ is necessary for neuronal survival and the developmental formation of the cerebellar cytoarchitectonics. As an end-result of decreased ER-TR mediation of hormone effects, functional deficiencies evolve later in life [17]. Thus, some of the developmental roles of ERβ are well established, little is known about the potential influential effects of the different EDs on cerebellar ERβ expression. To the best of our knowledge, no report thus far is available on the effect(s) of more EDs combined on cerebellar, or for that matter, any other cell type’s, ERβ expression.

To shed some light on this scientific puzzle, in the present study we deployed a frequently used a rat *in vitro* model that is widely accepted for experiments on neuronal development, neurogenesis, neurotoxicity and neuronal survival and apoptosis [5,18,19,20]. Primary rat cerebellar cell cultures were divided into two main groups: glia containing (Glia+) and “glia reduced” (Glia−) to test the effects of four known EDs, specifically bisphenol A (BPA), zearalenone (Zea), arsenic (As) and 4-methylbenzylidene camphor (MBC), individually or combined, on the expression level of ERβ mRNA. Both the *in vitro* experimental model (primary cerebellar cell culture) applied in this study [21] and the expression characteristics of ERβ [5] have been described previously in details. In addition, we recently demonstrated that the expression of ERβ (as well as that of TRs α and β) are regulated by both E2 and the TH triiodo-thyronine (T3). In the developing cerebellum, there is a highly complex interplay between E2 and THs in the maintenance of normal levels of each-other’s cognate receptors, and that the hormone effects are most probably mediated by the glia. Our observations implicate that abnormalities in glial and/or thyroid functions or in tissue E2/TH levels impact, on multiple levels, cerebellar development, cerebellar functions later in life, and the regenerative capability of the cerebellar tissue in case of injury, all of which should be considered in the diagnostics and treatment of relevant clinical conditions [22].

### Hypothesis

Endocrine disrupting chemicals, such as BPA, Zea, As and MBC, alone or in combination alter ERβ mRNA expression compared to non-treated control (ntC) and E2 plus T3 treated reference samples. We also hypothesize that the astroglia may play a role in the mediation of ED effects on ERβ mRNA expression.

## 2. Materials and Methods

### 2.1. Animals

Since neither previous studies nor our own results indicated gender differences in the developing rat cerebellum, both male and female Sprague-Dawley rat pups (body weight: 18–20 g; vendor: TOXI-COOP Zrt., Budapest, Hungary) were used in these studies. Timed pregnant Sprague-Dawley rats were obtained from the vendor at least four days before they gave birth. Animals were kept under standard laboratory conditions, with tap water and regular rat chow ad libitum in a 12-h light, 12-h dark cycle. The date of the pup’s birth was considered as postnatal day 0 (P0). Animals were used for granule cell preparation on their P7. Attention has been paid to sort the siblings into different treatment groups.

Following the guidelines established by the NIH, the use of animals was approved by the Animal Welfare Board at Szent Istvan University Faculty of Veterinary Sciences and were approved by the regional animal welfare authority (registry No: XIV-I-001/2201-4/2012).

### 2.2. Preparation of Primary Granule Cell Cultures

Primary cerebellar cultures were prepared as described (and patented) earlier [21] with modifications, as follows. Animals were sacrificed by quick decapitation and the cerebella removed. Cell cultures were prepared without enzymatic treatment and were maintained in serum- and steroid-free conditions as previously described [21]. Cell counting indicated that greater than 95% of the cells contained in these cultures were granule cell neurons (Glia reduced cultures). A detailed description of the seeding process and graphical illustration of the established cerebellar cell cultures, including the illustration of glia content, were as shown earlier [22]. Cerebella of rat pups were seeded into separate culture dishes (one dish from one pup, six dishes per treatment, *n* = 6). One dish contained around 21.3 × 10^6^ granule cells.

### 2.3. Treatments

For analysis of primary cerebellar granule cells in a glia reduced environment, a final concentration of 10 µM cytosine β-d-arabinofuranoside (AraC; Sigma Aldrich Ltd., Budapest, Hungary) was added 24 h after seeding to inhibit the proliferation of non-neuronal cells (Glia– experimental groups). In contrast, no AraC was added to the media for analysis of neurons grown in a regular glia containing environment (Glia+ experimental groups). Cultures were simultaneously treated with the following hormones (at physiologically relevant concentrations) and/or endocrine disruptors seven days after seeding and 6 h before harvesting: E2 (1.16 × 10^−10^ M, Sigma Aldrich Ltd., Budapest, Hungary); T3, (9.2 × 10^−10^ M, Sigma Aldrich Ltd., Budapest, Hungary); BPA (10^−10^ M, Sigma Aldrich Ltd., Budapest, Hungary); Zea (10^−10^ M); and As (10^−7^ M); MBC (6.3 × 10^−8^ M). In the case of BPA and ZEA, the carrier solvent was 0.1% dimethyl sulfoxide (DMSO).

**Figure 1:**
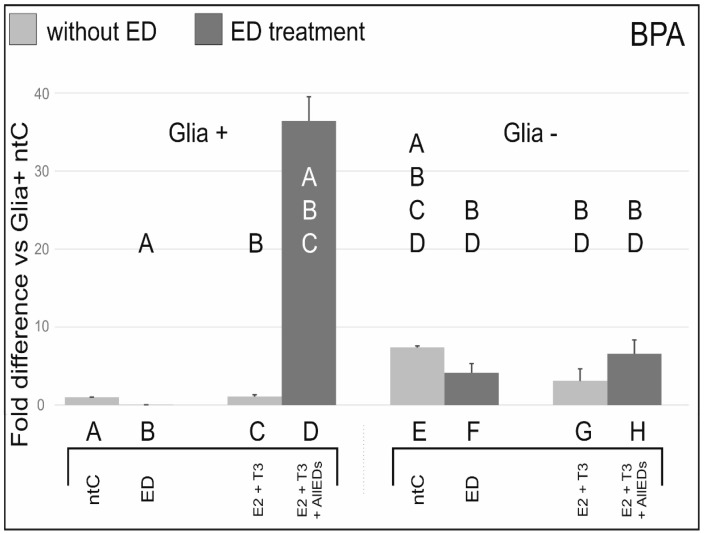
Effects of bisphenol A (BPA) with, and without, 17beta-estradiol (E2) and triiodo-thyronine (T3), on ERβ mRNA expression. BPA treatment decreased ERβ mRNA expression compared to the non-treated control. In glia-reduced cultures (Glia−), a more than two orders of magnitude higher (×180) ERβ mRNA expression was found in BPA treated cultures (column B *vs.* F). In glia containing samples (Glia+), there was about three magnitudes higher ERβ mRNA expression after E2 + T3 + BPA (column D) treatment than after BPA treatment alone (column B). Letters in and above the columns indicate significant differences (*p* ≤ 0.05) from columns below.

**Figure 2:**
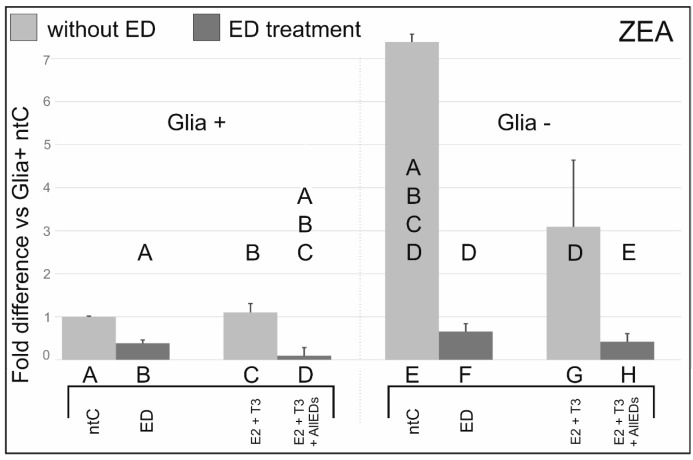
Effects of zearalenone (Zea) with, and without, 17beta-estradiol (E2) and triiodo-thyronine (T3), on ERβ mRNA expression. ERβ mRNA expression was substantially reduced by Zea treatment compared to the respective ntCs and Zea-void hormone treated cultures. Letters in and above the columns indicate significant differences (*p* ≤ 0.05) from columns below.

**Figure 3:**
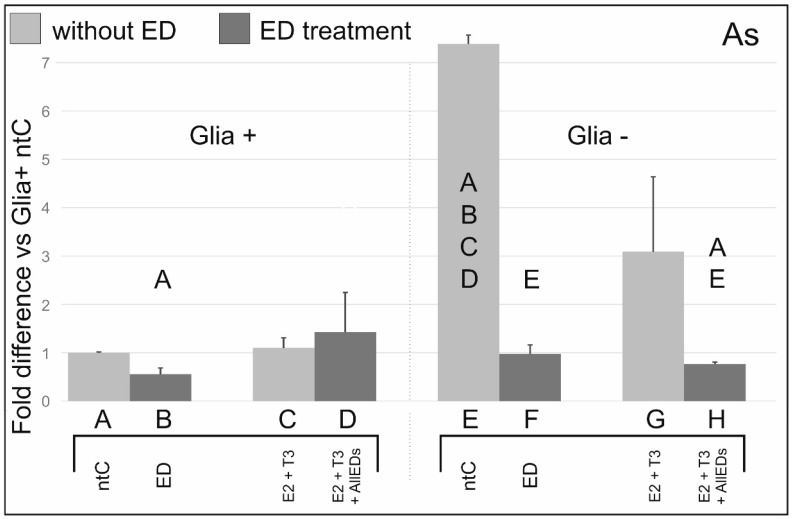
Effects of arsenic (As) with, and without, 17beta-estradiol (E2) and triiodo-thyronine (T3), on ERβ mRNA expression. ERβ mRNA expression levels after As and As + E2 + T3 treatments showed a pattern comparable in trends with that found in BPA studies in Glia+ and to those seen in Zea studies in Glia−. Letters in and above the columns indicate significant differences (*p* ≤ 0.05) from columns below.

**Figure 4:**
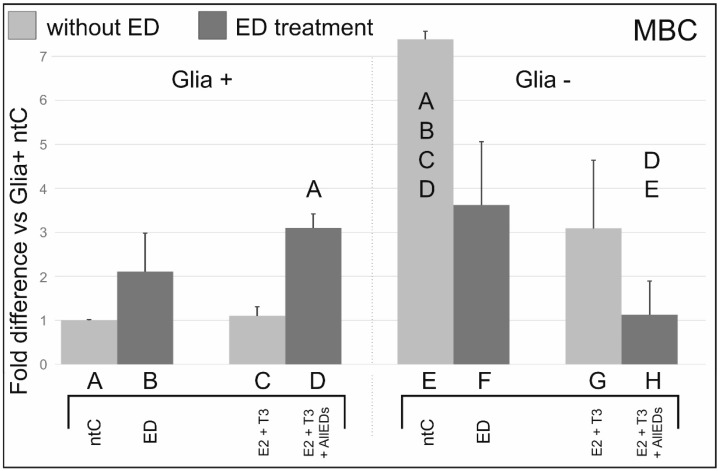
Effects of methylbenzylidene camphor (MBC) with, and without, 17beta-estradiol (E2) and triiodo-thyronine (T3), on ERβ mRNA expression. MBC tended to increase ERβ mRNA expression in Glia+ cultures. In Glia−, MBC decreased ERβ mRNA compared to its respective ntC, and E2 + T3 treated counterparts, although these differences did not reach significance. Letters in and above the columns indicate significant differences (*p* ≤ 0.05) from columns below.

**Figure 5:**
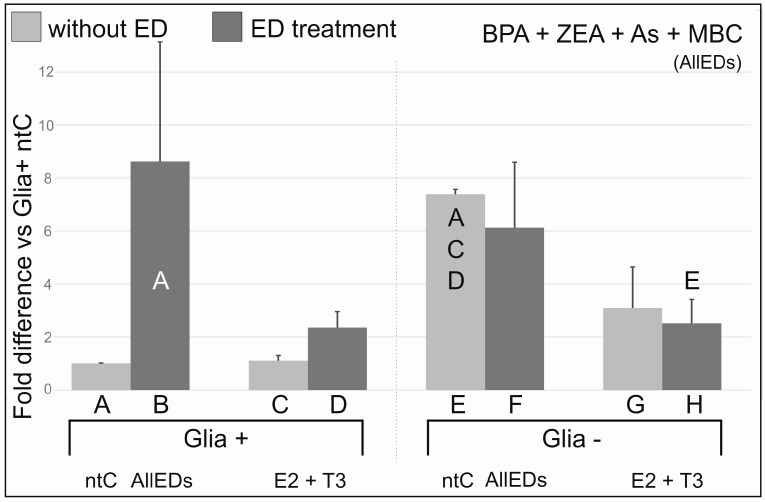
Effects of co-treatments with bisphenol A (BPA), zearalenone (Zea), arsenic (As), and methylbenzylidene camphor (MBC) with, and without 17beta-estradiol (E2) and triiodo-thyronine (T3) on ERβ mRNA expression. There was a robust and significant increase in ERβ mRNA expression in AllEDs treatment group of glia containing (Glia+) cultures, when compared to the ntC. However, high variance in differences were also found. In contrast, no increase was found in glia-reduced (Glia−) samples. ERβ mRNA expression values in E2 + T3 + All EDs groups were nearly identical in Glia+ *vs.* Glia− cultures. Compared to their respective non-treated controls, transcript levels in E2 + T3 + AllEDs cultures doubled in Glia+, while decreased to the one third in Glia−. Letters above the columns indicate significant differences (*p* ≤ 0.05) from columns below.

The applied concentration of the EDs was chosen based on our previous experiments [23], in which we tested BPA (as an estrogenic endocrine disrupting compound) effects *in vitro*, as follows: The biphasic effects of E2 and BPA at the low concentration range (10^−12^ to 10^−9^ M) were assumed to act at two binding sites, one stimulatory with a high affinity and one with a lower affinity that inhibits the effect of the first site. Based on the observed similarity in effect at each concentration it was also assumed that neither of the two sites distinguishes between E2 and BPA, and as of the present experiments, in the absence of relevant information, the same was assumed to all other EDs as well. Thus, data points were fit with the Hill-type equation:

(1) $$\text{Effect}=\text{maximal stimulation}\frac{1}{1+{\left(\frac{{\text{EC}50}_{\text{Stim}}}{\left[C\right]}\right)}^{H1}}\frac{1}{1+{\left(\frac{\left[C\right]}{{\text{EC}50}_{\text{Inhib}}}\right)}^{H2}}$$

where [C] is the concentration of the ligand. A best fit of the equation to the data resulted from setting the maximum effect of the stimulatory site (maximal stimulation) at 110%, with resulting values of 0.4 and 2 obtained for the stimulatory and the inhibitory Hill coefficients (H1 and H2), respectively. Under these conditions, EC50Stim and EC50Inhib were determined as 8 × 10^−12^ M and 4 × 10^−9^ M, respectively. Experimental groups were as displayed on Figure 1, Figure 2, Figure 3, Figure 4 and Figure 5.

### 2.4. Real-Time Quantitative PCR Measurements

Total RNA was isolated from the cell samples using TRIzol Reagent (Invitrogen, Carlsbad, CA, USA), according to the manufacturer’s instructions, and concentration were quantified by NanoDrop spectrophotometer ND-1000 (Thermo Scientific, Wilmington, DE, USA). Reverse transcription polymerase chain reaction was carried out as described by Sayed-Ahmed *et al.* [24]: 2 µg of total RNA was put together with 200 units of MMLV reverse transcriptase, 1 µg Oligo(Dt) and 10 mM dNTPs (final volume: 25 µL; for 60 min at 42 °C). Primer sequences (ERβ Forward TCCCAGCAGCAGTCAGTCCGA ERβ Reverse ACACCGCCACACAACCACCCT TM 60 size 400 bp) were as published by Vaillant *et al.* [25]. Cellular gene expression was quantified by quantitative PCR reactions (qPCR; LightCycler 2.0, F. Hoffmann-La Roche Ltd., Basel, Switzerland) using LightCycler DNA Master SYBRGReen I fluorescent dye (Hoffmann-La Roche Ltd., Basel, Switzerland). Aliquots of cDNAs were dispensed according to the manufacturer’s instructions. QPCR cycles and controls were planned according to the manufacturer’s instructions and were optimized for the primer pair. Amplified products were identified by agarose gel electrophoresis, melting point, and sequence analysis (Applied Biosystems ABI 3100 Genetic Analyzer, Agricultural Biotechnology Center, Gödöllő, Hungary). Real-time PCR threshold cycle (Ct) data were analyzed using the REST-XL software version 2.0 (developed by M. Pfaffl (Technical University Munich), Munich, Germany) [26]. The target Ct of each sample was normalized to the Ct of the reference gene (rat cytoplasmic beta actin) in the same sample. Differences in the Ct values were converted into relative amounts of mRNA based on the assumption that the amplification efficiency was 2.00. In the control group (ntC) mRNA value was arbitrarily set to 1 and results from other groups were expressed as fold changes relative to the control group.

### 2.5. Data Analysis

All data that have been presented are representative of at least three parallel independent measurements (from each sample). Statistical analyses were conducted using Excel (Microsoft, Microsoft Co., Redmond, WA, USA) and GraphPad Prism version 4 (GraphPad Software, San Diego, CA, USA), by means of one-way ANOVA with Tukey’s multiple comparison test. The level of statistical significance in differences between treatment groups are as shown in the figures (*p* ≤ 0.05). Statistical analyses were carried out by the Department of Biomathematics, Szent Istvan University Faculty of Veterinary Sciences.

## 3. Results

Results of hormone treatments were concordant with those we previously described [22], so they will not be the major focus of this report; rather, these non-ED results are used as the reference base for the comparison between the effects of EDs alone and in combination with E2 and T3. Additionally, as described in the aforementioned study, transcriptional activity was higher in ntC(Glia−) cultures compared to ntC(Glia+). Numerical values of the means and SEM values can be found as Table A1 in the Appendix section.

### 3.1. Bisphenol A

In Glia+, BPA alone reduced ERβ transcription to one third, whereas in Glia– there was only a minimal, non-significant reduction compared to the respective ntCs. Additionally, in Glia+, when BPA was co-administered with E2 + T3, ERβ mRNA expression increased 35-fold, while no effect of BPA + E2 + T3 co-administration was detected in Glia−.

### 3.2. Zearalenone

In Glia+, Zea administration to the culture medium decreased ERβ transcription three-fold, while Zea + E2 + T3 treatment resulted in ten-fold decrease compared to E2 + T3 treatment. In Glia−, ZEA treatment decreased ten-fold mRNA values compared to its respective ntC, while substantially, but not significantly, reduced mRNA expression in E2 + T3 + ZEA *vs.* E2 + T3.

### 3.3. Arsenic

In Glia+, As decreases ERβ mRNA expression to half of that detected in ntC; however, when co-administered with E2 + T3, a slight but not significant ERβ transcription increase was seen. In Glia−, both As and As + E2 + T3 treatments decreased ERβ mRNA expression levels approximately seven-fold compared to ntC(Glia−).

### 3.4. 4-Methylbenzylidene Camphor

In Glia+, both MBC and MBC + E2 + T3 treatment resulted in an increase in ERβ transcription, with a two-fold increase after MBC (not significant), and a three-fold increase after MBC + E2 + T3 treatment. This observation suggests that the action mechanism of MBC may differ from that of BPA, Zea and As. In Glia−, however, there was a two-fold decrease in ERβ mRNA levels after MBC- and a seven-fold decrease after MBC + E2 + T3 administration, reminiscent to what was found after As or As + E2 + T3 treatment.

### 3.5. AllEDs

In Glia+ AllEDs induced a 8.5-fold increase in the mean value of ERβ mRNA when cells were not co-exposed to E2 + T3 as well, and E2 + T3, co-administered with AllEDs doubled mean ERβ transcript values compared to E2 + T3-only treated samples. In Glia–, no significant change was detected after AllEDs treatment compared to ntC(Glia−); however, co-administration of AllEDs with E2 + T3 reduced ERβ mRNA to 1/3 of the respective ntC.

## 4. Discussion

The experimental model established from cerebellar cells (primary cerebellar cell cultures) has been extensively used over the past decades, both for testing of the cellular effects of different experimental cues in neurons and for the testing of the potential mediating role of the glia in those cellular effects [22]. Based on those studies, here we aimed to test whether four different EDs (BPA, Zea, As, and MBC) could influence ERβ mRNA expression with special emphasis on the potential effects after the co-administration of all four EDs with or without E2 and T3. Results of hormone treatments (without EDs) were reminiscent to those previously described [22]. Therefore, while we refer to those data when appropriate, below we are going to focus on the discussion of the effects of EDs alone and in combination with E2 and T3 on ERβ mRNA expression.

One of the questions that we should address is regarding the effect of glia in the adjustment of ERβ mRNA expression levels. In the present study, we compare endocrine effects in Glia+ (glia grown without growth-inhibitor) *vs.* Glia− (glia reduced) cultures. While differences between these groups is clearly the result of the presence or absence of glia, differences between treatment groups in Glia+ cultures carry the enigma of the share of glial ERβ in all cultured cell populations. Since, however, granule neurons extremely outnumber sporadic and rudimentary glial cells in Glia− cultures, it seems to be safe to interpret the treatment effects as if they were essentially exerted by neurons.

### 4.1. Effects of Bisphenol A

A continuously growing body of evidence indicates the adverse effects of BPA on health. A plethora of studies demonstrated that these BPA effects are mostly due to the estrogenic or anti-estrogenic potencies of this (and other) ED, as we have also demonstrated in the developing rat cerebellum *in vitro* [23], as well as *in vivo* [27]. The temporal and cellular appearance of ERβ in the cerebellum [2,5] suggests that EDs, including BPA, may act on both neurons and the glia to influence transcription through estrogen responsive elements and/or thyroid hormone responsive elements of relevant genes [22].

BPA’s effect to induce ERβ transcription in neurons appears to be downregulated by the glia. It should be noted, however, that such E2 and T3-deprived conditions can not be considered as physiological. Indeed, when BPA was co-administered with physiological concentrations of E2 and T3, results were significantly different from those found after BPA treatment alone, and even more so in glia-containing cultures. In Glia+, there was about three magnitudes higher ERβ mRNA expression after E2 + T3 + BPA treatment than after BPA treatment alone. Such combined treatment did not change ERβ mRNA expression significantly in glia reduced cultures. This observation suggests that BPA has an additive effect to E2 and T3 (non-published observation) and that the glia plays a role in the mediation of this effect. In summary, there is an apparent role of the glia in the mediation of BPA and BPA + E2 + T3 effects on ERβ transcription, and without this mediating role neither BPA nor BPA + E2 + T3 can induce a change in ERβ mRNA levels compared to the respective ntCs.

### 4.2. Effects of Zearalenone

To the best of our knowledge, there is no available information on the effects of Zea on cerebellar cells. There are, however, several studies indicating that Zea stimulates the cell proliferation in tumors of the female reproductive tract and breast malignancies in humans [28,29,30]. Neural cells are also obviously affected as shown in some sporadic reports. Zea clearly changes the expression of substances in the nerve fibers of the gastrointestinal such as vasoactive intestinal peptide, neuronal form of nitric oxide synthase, cocaine and amphetamine regulatory peptide, galanin, pituitary adenylate cyclase-activating peptide-27, and substance P [31]. Examining ovariectomized rats, Turcotte *et al.* [32] also reported that 2 mg of Zea injected subcutaneous induced the expression of neuronal progestin receptors comparable to that of 10 µg E2, and altered their sexual behavior, stimulating sexual receptivity. Early studies suggested that the biological effects of Zea may be due to their ability to bind to intracellular estrogen binding sites [33,34] Indeed, reporter gene assays demonstrated that Zea and its metabolites α- and β-zearalenol exhibit just slightly less strong estrogenic potency than 17-β estradiol itself [35]. Further study indicated that in the liver, Zea could also bind to cellular proteins distinct from estrogen receptors [36]. Kuiper *et al.* [37] found that Zea can bind to ERβ and can increase ERβ transcription. Our present findings show that mean values of ERβ mRNA expression levels are only higher in the comparison of Glia(−) *vs.* Glia(+) cultures. The finding that administration of Zea + E2 + T3 leads to significantly reduced ERβ transcription compared to Zea treatment alone in Glia+ suggests that, at least in the cerebellum, it is the absence of the natural hormones (E2 + T3), rather than the presence of Zea that increases ERβ transcription. Adding to the aforementioned idea, the lack of significance in the difference between E2 + T3 *vs.* E2 + T3 + ZEA in Glia− suggests that the determined hormone effects observed in Glia+ were exerted in glial cells. Altogether, the effects of Zea is the opposite of that of BPA in terms of the modification of the natural hormone effects in Glia+ *vs.* Glia− and, therefore, the effect of Zea is likewise mediated by the glia. Thus, it is possible that BPA and Zea may mask each other’s effects on ERβ transcription when cells are not exposed to other EDs.

### 4.3. Effects of Arsenic

Results in ERβ mRNA expression levels after As and As + E2 + T3 treatments showed a pattern comparable in trends with that found in BPA studies in Glia+ and to those seen in Zea studies in Glia−. It is noteworthy, however, that As + E2 + T3 treatments did not lead to such robust activation of ERβ transcription as detected after BPA + E2 + T3 treatments in Glia+. These observation raise the possibility that the effects of As may lie on the grounds of a mechanism distinct from that used by BPA.

Although, at present, we do not know the exact reason of the ERβ mRNA decreasing effect of As, it is possible that As may have caused a metabolic defect in cultured cells even during the relatively short, 6 h duration of the treatment.

Arsenic exposure leads to a multitude of neurodevelopmental dysfunction [38]. In children, cognitive function could irreversibly decline [39] and deleterious effects cause deficits in verbal and performance domains [40,41]. There are a number of reports describing neurotoxic effects of As on rodent pups resulting in sever decrease of locomotor activity and behavioral disorders [42]. Most studies, however, do not detail cellular and subcellular consequences that will be manifested in the above phenomena. Arsenic effects include increase lipid peroxidation leading to damages in plasma membrane and the intracellular membrane system. Furthermore, As inhibits elimination of free radicals, interferes in methylation reactions and in coupling with thiol groups, inducing DNA and protein damage [43,44,45].

It was Bodwell *et al.* [46] who first reported that As can act as ED on receptors for progesterone, androgen and corticoids in a dose-dependent manner and enhance hormone-dependent gene transcription even at very low doses [47]. ER were involved in these examinations later on, however demonstrating highly varying effects of As on ERβ expression. For example, Cimino-Reale *et al.* [48] reported that As increased ERβ expression in bone marrow cells. In contrast, Chen *et al.* [49] found that As only affected ERα, but not ERβ expression in breast carcinoma cells. Although information on As effects on ERβ is scarce, it appears that these As effects are tissue dependent. Our present results suggest that in the cerebellum, As can alter ERβ transcription, regardless of the absence or presence of glia. The question of whether possible interaction exists between As and one or more other EDs warrants further experiments.

### 4.4. Effects of 4-Methylbenzylidene Camphor

MBC, as an ED compound, affects the estrogenic and thyroid pathways as well [50,51,52,53], but the exact mechanism-of-action is still unknown. For example, a previous study demonstrated that MBC showed no binding to ERβ in human endometrial cells, nevertheless it affected ER mediated events [54]. Interestingly, however, Schlumpf *et al.* [51] found that MBC displaced 16-alpha-125-l-estradiol from ERβ, but not from ERα. Further studies also clarified that intrauterine exposure to MBC alters mRNA expression for both ERα and ERβ in uterine cells of the offspring [55]. A more detailed study by Schlumpf *et al.* [56] revealed that MBC exposure during the gestation affects ERα, ERβ, and progesterone receptor mRNA expression in various tissues, and also tissue-dependently, including the hypothalamus. MBC effects on the developing CNS is only a little known; moreover, those studies mostly focus on the hypothalamus-pituitary-gonad axis and the estrogen-related developmental changes in the early ages [52,57,58]. Adding to the afore-listed knowledge, probably the most prominent effect of MBC on our experimental model, among the other EDs used in this study, was its potency to increase ERβ mRNA expression in Glia+ cultures as compared to its respective ntC. Additionally, we detected an almost linear further increase in ERβ transcription if E2 + T3 were added to MBC that is consonant with previous studies stating that besides disrupting the estrogenic pathways, MBC also affects thyroid functions [50,56]. Our results suggest that the two endocrine effects might be synergistic that lies on the ground of a complex interplay between the estrogen and thyroid hormones and their receptors [22]. In Glia− cultures, however, MBC decreased ERβ mRNA compared to its respective ntC, with more decreasing potency when combined with E2 + T3. At the same time, it has to be noted that while mean values in our results are considerably different, variances were also high and, therefore, these differences were not statistically significant. Since, however, MBC may also affect thyroid functions [51], and its effects on different cell types seems to vary greatly, our results still remain alarming with regard to MBC’s influence on cerebellar development.

Altogether, of the EDs tested in this study, MBC appears to influence ERβ mRNA expression via a mechanism distinct from that/those involved in BPA-Zea-As effects; nevertheless, it is evident that MBC effects are also mediated by the glia.

In summarizing the results of the ED treatments, we detected changes in ERβ mRNA expression levels after each of the EDs that we used, and these results suggest that there are distinct ways how the EDs used can alter ERβ transcription. The observed versatility in ED effects, therefore, raised the question of how all the four EDs (AllEDs), co-added to the cultures, would affect ERβ mRNA, since in their everyday lives, living organisms are exposed to a variety of EDs simultaneously.

### 4.5. Effects of Treatments with BPA, Zea, As, and MBC Combined (AllEDs)

Since co-administration of E2 and T3 substantially reduced the robust effect of the mixture of EDs on ERβ transcription (Figure 2 column B *vs.* D), we conclude that under natural circumstances, when young individuals are simultaneously exposed to multiple EDs, their cerebellar development (and probably that of other CNS areas as well), is more affected by environmental EDs when secretion of T3 and E2 is insufficient. In addition it is reasonable to assume that high SEM values found in the AllEDs(Glia+) treatment group may be accounted for the individual susceptibility of the experimental animals for the EDs used in the treatments. Supporting this idea, E2 + T3, co-administered with AllEDs “only” doubled ERβ transcript values in Glia+. This may result from ERβ’s higher affinity to the hormone ligands.

ERβ mRNA expression values in AllEDs + E2 + T3 groups were nearly identical in Glia+ *vs.* Glia− cultures; therefore, it may appear that the glia did not play a role in the setting of ERβ transcript levels. However, compared to their respective ntCs, transcript levels doubled in Glia+, while decreased to the one third of the ntC value in Glia– in AllEDs + E2 + T3 treatment groups. Thus, one of the possible explanations is that the glia is needed to increase ERβ mRNA levels, however, it is also possible that under *in vitro* conditions and in the presence of the compounds used for these treatments, measured values represent an optimal ERβ transcript level for survival that can be set in both major cell types, neurons and glia, as well.

## 5. Conclusions

Thus, three important conclusions can be drawn: (1) EDs’ influential effects on ERβ transcription depends on the specific ED; (2) the glia modulates (mediates?) ED effects on the ERβ mRNA level in the cultured cell populations, and most probably the neuronal ERβ transcription as well; and (3) combined ED effects are exerted when E2 and T3 levels are low. The latter suggests that simultaneous ED (AllEDs) might have their highest impact in hypothyroid females in menopause.

## Abbreviations

The following abbreviations are used in this manuscript:

AllEDs	all the four Endocrine Disruptors
As	Arsenic
BPA	Bisphenol A
ED	Endocrine Disruptor
ER	Estrogen Receptor
E2	17β-estradiol
Glia+	glia containing cell culture
Glia–	glia reduced cell culture
MBC	4-methylbenzylidene camphor
ntC	non-treated control
P(0)	postnatal day 0
TH	thyroid hormone
TR	TH receptors
T3	Triiodo-thyronine
ZEA	Zearalenone

## Appendix

**Table A1:** Fold difference values (mean and SEM) from the different ED experimental groups.

	Glia+				Glia−			
	ntC	ED	E2 + T3	E2 + T3 + ED	ntC	ED	E2 + T3	E2 + T3 + ED
**BPA**								
MEAN	1000	0.023	1103	36,428	7387	4141	3093	6565
SEM	0.017	0.005	0.205	3087	0.182	1172	1546	1770
**ZEA**								
MEAN	1000	0.389	1103	0.096	7387	0.659	3093	0.421
SEM	0.017	0.073	0.205	0.190	0.182	0.182	1546	0.190
**As**								
MEAN	1000	0.559	1103	1428	7387	0.975	3093	0.769
SEM	0.017	0.129	0.205	0.819	0.182	0.188	1546	0.039
**MBC**								
MEAN	1000	2111	1103	3097	7387	3623	3093	1130
SEM	0.017	0.869	0.205	0.323	0.182	1436	1546	0.761
**AllEDs**								
MEAN	1000	8623	1103	2351	7387	6125	3093	2522
SEM	0.017	4520	0.205	0.610	0.182	2468	1546	0.894
